# The effects of traditional games on physical literacy among school-aged children

**DOI:** 10.3389/fspor.2025.1759890

**Published:** 2026-01-16

**Authors:** Meliha Çinar, Fahimeh Hassani

**Affiliations:** Department of Physical Education, Istanbul Gedik University, Istanbul, Türkiye

**Keywords:** active lifestyles, culturally rooted play, movement skills, physical competence, physical literacy, traditonal game

## Abstract

**Introduction:**

Physical literacy is crucial for promoting lifelong engagement in physical activity. In response to rising childhood inactivity, this study explores the impact of traditional children's games on physical literacy within a school context.

**Methods:**

A quasi-experimental design was applied involving 60 students (aged 11–12) from two schools in Trabzon, Turkey. The experimental group participated in an 8-week program of culturally-rooted traditional games. The control group followed standard physical education activities. Pre- and post-test data were collected using a validated Physical Literacy Scale.

**Results:**

Statistical analyses indicated significant improvements (*p* < .001) in all four domains of physical literacy—physical, psychological, social, and cognitive—in the experimental group compared to the control group.

**Discussion:**

Traditional games are shown to be inclusive, culturally relevant, and effective pedagogical tools for improving physical literacy in school-aged children. Their integration into school curricula is supported as a cost-effective and holistic educational strategy.

## İntroduction

Play, one of the most fundamental elements in human development from early childhood, has roots that date back centuries. Among the many types of play passed down through generations, traditional children's games hold a unique and invaluable place. These games not only support the physical development of children, but also contribute to their social, emotional, and cognitive growth. Moreover, they serve as a form of cultural heritage, reflecting a society's history, traditions, values, and way of life. The continuation of these traditional games helps instill cultural identity in children and shapes them into more socially connected individuals (3). Despite their developmental and cultural value, traditional children's games have been increasingly replaced by sedentary behaviors, digital entertainment, and structured academic demands. This decline in traditional play has raised serious concerns regarding children's movement opportunities and overall physical development (4). The increasing physical inactivity observed in children has underscored the need for a more pedagogically comprehensive approach to physical education. Physical literacy, which embodies an active lifestyle and healthy living philosophy within schools, has emerged as a core component of physical education. It is recognized as a critical framework for enhancing overall health and well-being (5).

Physical literacy is defined as the combination of motivation, confidence, physical competence, knowledge, and understanding that empowers individuals to value and take responsibility for engaging in physical activities throughout life (6). Physical literacy enables individuals to maintain purposeful and lifelong physical activity by fostering competence, motivation, and confidence (7). In addition to developing a variety of personal skills, physical literacy plays a pivotal role in promoting a healthy quality of life. Acquiring these skills from early childhood, particularly from preschool age, is a vital step toward lifelong healthy development (8). However, recent evidence indicates that many school-age children demonstrate low levels of physical literacy, particularly in movement competence and motivation for physical activity, suggesting that current physical education programs may be insufficient in addressing children's holistic developmental needs (1, 9). Therefore, fostering physical literacy is essential to ensure individuals lead active lives throughout their lifetime. However, formal physical education classes alone may not suffice in developing these skills. Traditional children's games present a promising pedagogical approach, as they naturally integrate fundamental movement skills, social interaction, emotional regulation, and enjoyment within meaningful cultural contexts (10). It is equally important to integrate traditional children's games into students' routines as a valuable means to reinforce learning and utilize leisure time effectively (10). The dynamic and cooperative structure of traditional games supports the development of movement competence through repetitive motor actions, enhances social–emotional skills via collaboration and rule-based interaction, and fosters intrinsic motivation by providing enjoyment and autonomy in physical activity participation (1, 11). In today's modern educational systems, where technology often reduces children's movement opportunities, the revival and integration of traditional games can provide an engaging and culturally meaningful solution.

Despite growing international interest in physical literacy, there remains a notable research gap regarding the empirical examination of traditional children's games as structured interventions to enhance physical literacy, particularly within school-based settings and specific cultural contexts such as Turkey (2). Addressing this gap is crucial, as culturally relevant and low-cost strategies are increasingly needed to counteract physical inactivity and disengagement from physical education among children.

Therefore, the present study aims to investigate the effectiveness of traditional children's games in enhancing physical literacy among school-age children. By responding to current educational challenges and addressing an underexplored area in the literature, this research seeks to contribute meaningful evidence to support culturally grounded, play-based approaches in physical education.

## Method

### Research design

This study employed a quantitative research method with a pre-test–post-test control group experimental design. The sample was divided into male and female students, forming two groups: one experimental (intervention) and one control group. Both groups were given the same survey instruments before and after the intervention. The absence of statistically significant differences between the groups across all variables was considered a criterion for homogeneity. This quasi-experimental design was selected due to its suitability for educational field interventions where random assignment is not always feasible (12). It allowed for the evaluation of intervention effects by comparing pre- and post-test differences across matched groups.

### Population and sample

The study was conducted during the 2024–2025 academic year and targeted students from Kadıköy and Kerem Secondary Schools, located in the Çarşıbaşı district of Trabzon, under the supervision of the Ministry of National Education. The sample consisted of 60 students aged 11–12, including 30 boys and 30 girls. Each of the experimental and control groups included 15 boys and 15 girls, ensuring gender balance. The control group was selected from a village school, similar in profile to the experimental group, to ensure environmental consistency*.* Simple random sampling was used to reduce selection bias and ensure representativeness of the sample population (13).

All participants were selected using a simple random sampling method, and necessary permissions were obtained from school administrators, parents, and the Provincial Directorate of National Education in Trabzon. As the participants were minors, written informed consent was obtained from their parents or legal guardians prior to participation. A dedicated information session was held with the parents to explain the study's aims, procedures, and ethical considerations in detail. Only participants whose parents or guardians provided written consent were included in the research.

### Data collection tools

The data collection instrument used in this study was the “Physical Literacy Scale in Children,” originally developed by (1) and later adapted and validated for older children by (2). This tool was chosen due to its multidimensional approach to physical literacy, covering physical, psychological, social, and cognitive domains, which aligns with the study's holistic conceptual framework. The scale includes four sub-dimensions: physical, psychological, social, and cognitive, comprising a total of 30 items evaluated on a 4-point Likert scale. The scale measures how children perceive themselves using statements like “very much like me” or “a bit like me,” selecting the descriptor that best fits. The scoring ranges are: Physical (12–48), Psychological (7–28), Social (4–16), and Cognitive (7–28). Higher total scores indicate greater levels of perceived physical literacy. The scale's reliability and validity have been confirmed for Turkish populations (2).

### Procedure

Before the intervention, both groups completed a pre-test. The experimental group (Kadıköy Secondary School) participated in an 8-week traditional games program held in the schoolyard under the supervision of a physical education teacher. The control group (Kerem Secondary School) did not participate in any traditional games. After the intervention, both groups completed a post-test. The intervention was designed to actively target movement competence, motivation, social interaction, and cognitive engagement, four key components of physical literacy—through structured traditional games. The program included:

Castle Dodgeball (Kaleli Yakan Top): A team game where players defend a designated “castle” while trying to eliminate opponents with a ball.
Handkerchief Grab (Mendil Kapmaca): Two players race to grab a handkerchief placed in the middle and return to their team without being tagged.
Nine Stones (Dokuztaş): Players knock down a pile of nine stones with a ball, then attempt to rebuild it while avoiding being hit.
Hopscotch (Sek Sek): Players hop through a numbered pattern on one foot, retrieving a marker without stepping outside the lines.
Sack Race (Çuval Yarışı): Participants race by hopping inside a sack toward the finish line.
Tug of War (Halat Çekme): Two teams pull on opposite ends of a rope, aiming to drag the other team past a line.
Tangle Game (Arapsaçı): Players hold hands in a circle, twist to create a tangle, then cooperate to untangle without letting go.
Corner Tag (Köşe Kapmaca): Players compete for limited corner spaces while avoiding being tagged.

The selection of these games was based on their culturally relevant nature, ability to foster inclusive participation, and alignment with developmental goals for school-aged children (10).

### Data analysis

All collected data were analyzed using SPSS version 22.0. To determine whether the data followed a normal distribution, the Shapiro–Wilk test was employed. Since the data were not normally distributed (*p* < 0.05), the Mann–Whitney *U* test was used to compare differences between the experimental and control groups, while the Wilcoxon Signed-Rank test was used to assess changes between pre-test and post-test scores. Statistical significance was considered at *p* < .05. Non-parametric tests were deemed appropriate based on data characteristics and sample size (14).

## Results

Descriptive statistics for participant demographics are presented in Table 1. The sample consisted of 30 students evenly divided into experimental and control groups, each including 15 boys and 15 girls. The participants were either 11 or 12 years old. In the control group, 14 students were aged 11 (M = 11.00, SD = 0.00), and 16 were aged 12 (M = 12.00, SD = 0.00). The experimental group had a similar age distribution: 14 students were aged 11 (M = 11.00, SD = 0.00), and 16 were aged 12 (M = 12.00, SD = 0.00). The overall average age in both groups was 11.53 years (SD = 0.50).

**Table 1:** Participants' average age, standard deviation, and gender distribution.

Group		Control			Experiment		
Gender	Boy	15			15		
	Girl	15			15		
Age		Number	Mean	S. S	Number	Mean	S. S
	11	14	11.00	0.00	14	11.00	0.00
	12	16	12.00	0.00	16	12.00	0.00
	Total	30	11.53	0.50	30	11.53	0.50

The descriptive statistics are shown in Table 1.

According to Table 2, there was no statistically significant difference in the pre-test scores of students between the control and experimental groups in the dimensions of physical literacy (physical, psychological, social, cognitive). The analysis showed no significant differences in the pre-test scores between the control and experimental groups across all dimensions of physical literacy. The post-test analysis revealed statistically significant differences between the control and experimental groups across all dimensions of physical literacy (physical, psychological, social, and cognitive). These differences were deemed statistically significant as the *p*-values were less than.05. This indicates that there were meaningful differences in post-test scores between the control and experimental groups.

**Table 2:** Combined pre-test and post-test comparison of physical literacy dimensions between control and experimental groups.

Test Type	Dimension	Mann–Whitney U	Z Score	*p* Value
Pre-Test	Physical	360	−1.32	0.185
	Psychological	303	−0.84	0.390
	Social	358	−1.37	0.170
	Cognitive	432	0.63	0.520
	Physical Literacy	432	−0.26	0.790
Post-Test	Physical	73.00	−5.58	0.000
	Psychological	28.00	−6.28	0.000
	Social	85.50	−5.49	0.000
	Cognitive	34.00	−6.24	0.000
	Physical Literacy	19.00	−6.37	0.000

The Wilcoxon Signed-Rank test revealed statistically significant improvements across all dimensions of physical literacy within the experimental group. Specifically, significant differences were found in physical (Z = −4.19, *p* < .001), psychological (Z = −4.30, *p* < .001), social (Z = −4.53, *p* < .001), and cognitive dimensions (Z = −4.22, *p* < .001), as well as in overall physical literacy (Z = −4.60, *p* < .001). These results indicate that the intervention had a positive effect on the physical literacy levels of students in the experimental group. The results also indicated statistically significant differences between pre-test and post-test scores within the control group. Improvements were observed in the physical (Z = −2.78, *p* = .005), psychological (Z = −4.19, *p* < .001), social (Z = −3.04, *p* = .002), and cognitive (Z = 4.42, *p* < .001) dimensions, as well as in overall physical literacy (Z = 4.31, *p* < .001). Although these results show that the control group experienced statistically significant changes, the direction and magnitude of the Z-scores suggest that the experimental group achieved greater improvements following the intervention (Table 3).

**Table 3:** Comparison of physical literacy within the experimental and control groups*.*

Group	Dimension	Z Score	*p* Value
Experimental	Physical	−4.186	0.000
	Psychological	−4.295	0.000
	Social	−4.529	0.000
	Cognitive	−4.216	0.000
	Overall Physical Literacy	−4.599	0.000
Control	Physical	−2.780	0.005
	Psychological	−4.191	0.000
	Social	−3.039	0.002
	Cognitive	4.415	0.000
	Overall Physical Literacy	4.314	0.000

## Discussion

The results of this study support the positive impact of traditional children's games on the physical literacy of students. The findings reveal significant improvements in all four dimensions of physical literacy—physical, psychological, social, and cognitive—among the experimental group that participated in traditional games. These improvements align with previous research indicating that physical literacy is not only a reflection of physical skills but also incorporates psychological, social, and cognitive dimensions (7, 15).

Traditional games, as enjoyable physical activities with moderate to low intensity, can have numerous positive effects on children's physical literacy and cognitive development. These games not only enhance physical abilities but also play a significant role in supporting emotional and mental well-being. When incorporated into physical education programs, traditional games help stimulate creativity, strengthen decision-making skills, and promote social interaction, thus contributing to children's holistic development. In support of this, Hassani and Aydın highlight the challenges in designing physical activity programs that effectively improve physical literacy. Their research underlines the importance of creating programs that address the diverse needs of children by considering both their cognitive and emotional development (16). They emphasize that activities should strike a balance between simplicity and complexity—simple enough to avoid frustration, yet complex enough to maintain interest and prevent boredom (16). Furthermore, these activities must be engaging and enjoyable to sustain motivation and active participation (11). Traditional games meet these pedagogical criteria by offering flexible, culturally familiar, and socially engaging activities that promote not only physical competence but also emotional resilience and peer collaboration (17). Traditional games naturally fulfill these criteria. They offer adjustable levels of difficulty, ensuring they are neither too easy nor overwhelming. Their interactive and culturally rooted nature keeps children engaged while fostering a sense of enjoyment and connection. Additionally, traditional games encourage teamwork, communication, and emotional regulation—elements that are essential for comprehensive physical literacy. As such, they serve not only as tools for physical engagement but also as effective educational strategies that align with modern principles of inclusive program design.

Traditional games offer athletic advantages that align with the cultural, artistic, and regional characteristics of various communities, making them effective tools for providing healthy and meaningful physical activities (10). These culturally rooted games not only enhance physical literacy but also increase physical activity levels, a key component of physical literacy (18). In comparison, while the control group also showed improvements, the changes were less pronounced—highlighting the dynamic and engaging nature of traditional games as superior to standard physical education practices in schools (19). Moreover, children participating in traditional games often demonstrate higher levels of motivation and willingness to participate in physical activity, which is critical for sustaining healthy behavior patterns (20); (Whitehead, 2013). Integrating such games into the school curriculum can expose students to a broader array of experiences that foster physical competence, confidence, motivation, and understanding, as supported by the International Physical Literacy Association (21). Moreover, traditional games carry substantial social benefits that contribute to holistic student development. These games naturally promote ethical behavior, such as fairness, respect for rules, and mutual respect among participants (22). Their inherently interactive structure fosters peer collaboration, empathy, and effective communication, strengthening interpersonal relationships and emotional intelligence (10). Through cooperative play, children learn essential social skills such as turn-taking, conflict resolution, and working towards common goals—skills that are foundational for responsible citizenship and community engagement (19). The inclusive nature of traditional games nurtures a strong sense of belonging, unity, and shared cultural identity, reinforcing the value of community and societal norms (18). Drawing from theories of social development and mirror neuron research, playing in group settings allows children to internalize pro-social behaviors and strengthen their social cognition (10). As students engage in culturally familiar and socially meaningful play, they not only build physical competencies but also develop ethical awareness, cultural sensitivity, and a deeper appreciation for diversity. This makes traditional games a powerful educational medium for connecting physical education to broader social and cultural learning outcomes, ultimately enhancing both individual growth and social cohesion. The playful and cooperative nature of these games may also explain the improvement in psychological and social dimensions of physical literacy, as children develop communication skills, teamwork, and a sense of belonging (8).

In addition to the physical, social, and cognitive benefits already outlined, traditional games also significantly contribute to the psychological aspects of physical literacy, particularly in enhancing self-motivation, self-regulation, self-confidence, and self-concept in children. As Piaget's theory of development suggests, play is intrinsically connected to emotional and cognitive growth. Unstructured and enjoyable games foster intrinsic motivation by providing autonomy and a sense of competence (11). Traditional games, being non-pressured and outcome-oriented, offer repeated opportunities for success in a social context, which nurtures a child's sense of self-efficacy and encourages continued participation in physical activities (23). Moreover, through engagement in traditional games, children learn to regulate their emotions, delay gratification, and navigate social dynamics—skills essential for self-regulation (24). The emotional fluctuations experienced during cooperative and competitive play—such as excitement, disappointment, and joy—build resilience and emotional intelligence. These emotional and cognitive engagements lead to the development of a healthy self-concept, where children view themselves as competent, capable, and valued members of a community. Importantly, motivation plays a cyclical role in this developmental process: it promotes engagement, which enhances competence, builds self-esteem, and ultimately reinforces intrinsic motivation (20). Thus, the inclusion of traditional games in educational curricula not only improves physical literacy but also supports the psychological foundations necessary for lifelong participation in physical activity. This psychological development directly contributes to sustained physical activity and overall well-being, reinforcing the cyclical relationship between motivation, competence, and lifelong physical literacy (25). This study is limited by its quasi-experimental design, relatively small sample size, and short intervention duration. These factors may restrict the generalizability of the findings. Future research should consider employing randomized controlled trials with larger and more diverse populations, as well as extended follow-up periods, to examine the long-term impact of traditional games on physical literacy.

## Conclusion

This study demonstrated that an eight-week traditional games–based intervention resulted in statistically significant improvements in children's physical literacy across physical, psychological, social, and cognitive domains. The findings indicate that structured traditional games are effective pedagogical tools for enhancing movement competence, motivation for physical activity, and socio-emotional development among school-aged children in formal educational settings. From an applied perspective, the results support the integration of culturally grounded and play-based activities into physical education curricula as low-cost and inclusive strategies for promoting holistic physical literacy. Given the quasi-experimental design and limited sample size, future research should focus on longitudinal investigations to assess the sustainability of these outcomes and examine the effectiveness of traditional games in inclusive educational contexts, particularly for children with special educational needs.

## Data Availability

The datasets presented in this article are not readily available because The dataset used in this study contains information collected from minors and is therefore subject to ethical and privacy restrictions. Data cannot be publicly shared due to confidentiality agreements outlined in the institutional ethics approval and informed consent procedures. De-identified or aggregated data may be made available upon reasonable request to the corresponding author, pending approval from the relevant ethics committee. Requests to access the datasets should be directed to Fahimeh.hassani@gedik.edu.tr.
